# Effects of bisphenol F, bisphenol S, and bisphenol AF on cultured human osteoblasts

**DOI:** 10.1007/s00204-023-03523-2

**Published:** 2023-05-17

**Authors:** E. García-Recio, V. J. Costela-Ruiz, L. Melguizo-Rodríguez, J. Ramos-Torrecillas, R. Illescas-Montes, E. De Luna-Bertos, C. Ruiz

**Affiliations:** 1 Biomedical Group (BIO277), Department of Nursing, Faculty of Health Sciences, University of Granada, Avda. Ilustración 60, 18016 Granada, Spain; 2 Institute of Biosanitary Research, ibs.Granada, Avda. de Madrid, 15 Pabellón de Consultas Externas, 2ª Planta, 18012 Granada, Spain; 3 Institute of Neuroscience, University of Granada, 18016 Granada, Spain

**Keywords:** Bisphenol F, Bisphenol S, Bisphenol AF, Osteoblast, Cellular viability, Cellular differentiation

## Abstract

Bisphenol A (BPA) analogs, like BPA, could have adverse effects on human health including bone health. The aim was to determine the effect of BPF, BPS and BPAF on the growth and differentiation of cultured human osteoblasts. Osteoblasts primary culture from bone chips harvested during routine dental work and treated with BPF, BPS, or BPAF for 24 h at doses of 10^–5^, 10^–6^, and 10^–7^ M. Next, cell proliferation was studied, apoptosis induction, and alkaline phosphatase (ALP) activity. In addition, mineralization was evaluated at 7, 14, and 21 days of cell culture in an osteogenic medium supplemented with BP analog at the studied doses. BPS treatment inhibited proliferation in a dose-dependent manner at all three doses by inducing apoptosis; BPF exerted a significant inhibitory effect on cell proliferation at the highest dose alone by an increase of apoptosis; while BPAF had no effect on proliferation or cell viability. Cell differentiation was adversely affected by treatment with BPA analogs in a dose-dependent, observing a reduction in calcium nodule formation at 21 days. According to the results obtained, these BPA analogs could potentially pose a threat to bone health, depending on their concentration in the organism.

## Introduction

Exposure to bisphenol A (BPA) has been associated with numerous diseases (e.g., cancer, obesity, and reproductive health disorders) due to its action as an endocrine disruptor (Adoamnei et al. 2018; Pelch et al. 2019; Ma et al. 2019; den Braver-Sewradj et al. 2020). This highly ubiquitous toxin is found in a wide range of items of daily use, being commonly employed in the manufacture of containers, utensils, and food packaging, among many other products (Staples et al. 1998; Huang et al. 2012; Abraham and Chakraborty 2020). Hence, humans are in virtually continuous contact with this molecule (Fromme et al. 2002; Abraham and Chakraborty 2020). Evidence of the elevated toxicity and environmental ubiquity of BPA has prompted measures aimed at its elimination. These included the prohibition by the European Commission of its utilization in baby bottles (European Commission 2011) and thermal paper (European Commission 2016). A recent European Food Safety Authority (EFSA) draft opinion has proposed to lower the tolerable daily intake of BPA from 4 μg/kg/day to 0.04 ng/kg/day, therefore potential health risks need to be addressed (EFSA 2021). Legislative action and consumer pressure have led to the widespread replacement of BPA by its analogs, including BPF, BPS, and BPAF. However, these have been reported to have comparable endocrine-disrupting effects to those of BPA, with similar possible health repercussions (Rochester and Bolden 2015; Chen et al. 2016; Rosenfeld 2017).

Endocrine disruptors can interfere with bone homeostasis by causing a hormonal imbalance, by exerting a direct toxic effect on osteoblasts, or by triggering osteoclastic activity (Yaglova and Yaglov 2021). Estrogens play a major role in bone tissue regulation mechanisms, and bisphenols act as xenoestrogens. They can therefore be responsible for hormonal imbalances with potential repercussions for the structural and functional properties of bone tissue (Chin et al. 2018). Various studies have reported that cell physiology is altered by the interaction of BPA with the estrogenic and xenobiotic receptors of osteoblasts and osteoclasts (Bolli et al. 2008; Vrzal et al. 2015; Thent et al. 2018; Giannattasio et al. 2021; Wang et al. 2021). However, there has been scant research on the effect of BPA on osteoblasts and studied cell populations have been varied, including murine osteoblasts (Hwang et al. 2013), human fetal osteoblasts (Thent et al. 2018) and, very recently, human osteoblasts obtained by primary culture (García-Recio et al. 2022). In this last study, BPA inhibited the proliferative capacity of osteoblasts at doses ranging from 10^–5^ M to 10^–7^ M by inducing apoptosis. It also inhibited mineralization by reducing alkaline phosphatase (ALP) synthesis and the consequent formation of calcium nodules. At a molecular level, BPA and its analogs (BPF, BPS, and BPAF) inhibited the gene expression of osteogenic markers closely related to osteoblastogenesis and osteoblast function, i.e., RUNX2, OXS, OSC, ALP, COL-1, BMP-2, and BMP-7 (García-Recio et al. 2022, 2023).

The objective of this in vitro study was to determine the effect of BPF, BPS, and BPAF on the growth and differentiation of human osteoblasts.

## Materials and methods

### Chemical

BPF, BPS and BPAF supplied by Sigma-Aldrich (St Louis, MO, USA), supplied by Sigma-Aldrich (Co., MO), was dissolved in dimethyl sulfoxide (DMSO). The final DMSO concentration was always ≤ 0.05%.

### Primary human osteoblasts

Primary human osteoblasts were taken from bone chips harvested during routine mandibular osteotomy or lower wisdom tooth extraction in healthy individuals at the Clinic of the School of Dentistry of the University of Granada. Three patients were recruited for this trial from which three primary human osteoblasts cell lines were established. Each cell line was cultured independently. All participants signed informed consent to participate in the study.

### Treatments

The osteoblast cells obtained were treated for 24 h with BPF, BPS, or BPAF at doses of 10^–5^ M, 10^–6^ M, or 10^–7^ M; untreated cells were used as controls.

### Cell proliferation assay

Cell proliferation was determined by MTT colorimetric assay (3-(4,5-dimethylthiazol-2-yl)-2,5-diphenyltetrazolium bromide) (Sigma) as described Illescas-Montes et al., 2017. In brief, cells were cultured in 96-well plates at a concentration of 1 × 104 cells/mL and were synchronized for 24 h in DMEM supplemented with 2% FBS. After treatment, cells were added to a medium without phenol red and with MTT and incubating them for 4 h at 37ºC in a humidified atmosphere (95% air 5% CO2). After incubation, the formazan crystals were dissolved by adding dimethyl-sulfoxide, and the absorbance was measured at 570 nm using a spectrophotometer (SunriseTM, Tecan, Männedorf, Switzerland). The results were expressed as a percentage of the absorbance with respect to the control group.

### Apoptosis and necrosis analysis

Cultured human osteoblast cells treated with different BPs at a concentration of 10^–5^ M, 10^–6^ M, or 10^–7^ M for 24 h and untreated control cells. Apoptosis and necrosis were studied as described (Costela-Ruiz et al. 2019). The results were expressed as the percentage of cells annexin-positive (apoptotic) and propidium iodide-positive (necrotic).

### ALP activity

Primary human osteoblasts were grown to the confluence with a culture medium supplemented with 10 mM β- glycerophosphate and 50 μg/mL of ascorbic acid to stimulate differentiation. After 6 days, cells were incubated with doses 10–7 to 10–5 M of BPS, BPF and BPAF for 48 h. Cells were lysed with 0.1% Triton X-100 as described by (Melguizo-Rodríguez et al. 2018). Untreated cells were used as control. ALP activity was quantified with a colorimetric assay (Diagnostic kit 104-LL, Sigma, St. Louis, MO) using p-nitrophenyl phosphate as a substrate, as described Garcia-Recio et al. 2022. Total protein content was estimated by the Bradford method. The results of each assay were compared with those for untreated cells grown under the same conditions and expressed as a percentage of U/mg protein in relation to a control group.

### Mineralization assay

Cells were cultured in DMEM with ascorbic acid (0.05 mM) and β-glycerol phosphate (5 mM), supplemented with the different doses (10–5, 10–6, or 10–7 M) of BPs tested or unsupplemented (control group). The plate with cells and precipitated calcium added to the cell matrix was stained with alizarin red S (2%) at 7, 14, and 21 days. After extraction of the dye present in mineralization nodules for 15 min with 10% (w/v) cetylpyridinium chloride in 10 mM sodium phosphate (pH 7.0), the extracted stain was transferred to a 96-well plate, and the absorbance was measured at 562 nm with a spectrophotometer (ELx800, Biotek) as previously reported (García-Recio et al. 2022).

## Results

### Effect of BPF, BPS, and BPAF on osteoblast growth

The impact of BPF, BPS, and BPAF on osteoblast growth was evaluated by the study in parallel with their effects on cell proliferation and apoptosis/necrosis induction.

**Fig. 1:**
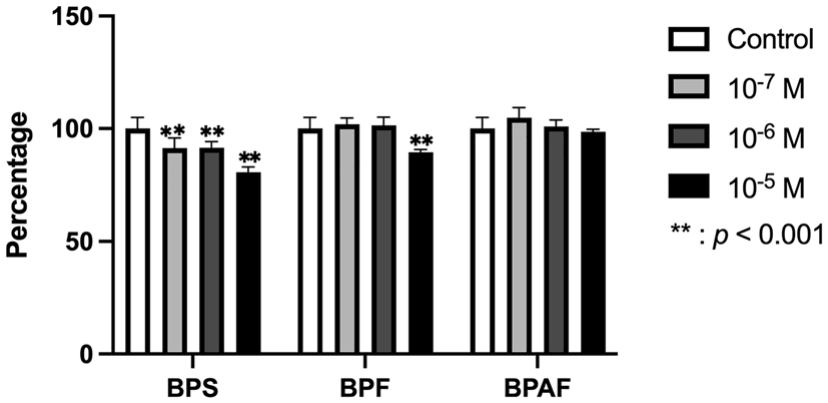
Effect of different doses (10^–5^ M, 10^–6^ M, or 10^–7^ M) of BPS, BPF, or BPAF on osteoblast proliferation in primary cell line after 24 h of incubation. Data are expressed as means ± standard deviation

Figure 1 depicts the data obtained for human osteoblast proliferation after 24 h of culture in the presence of 10^–5^, 10^–6^, or 10^–7^ M of the studied bisphenols. The proliferative capacity of osteoblasts was significantly inhibited after BPS treatment at all three doses (*p* ≤ 0.001) and after BPF treatment at the highest dose (10^–5^ M) (*p* ≤ 0.001), but it was not significantly affected by BPAF treatment at any dose.

The results of the apoptosis/necrosis study are exhibited in Table 1. BPS treatment at doses of 10^–5^, 10^–6^, and 10^–7^ M significantly increased the percentage of apoptotic cells but did not affect the percentage of necrotic cells. BPF treatment significantly increased the percentage of apoptotic cells at a dose of 10^–5^ M alone (*p* ≤ 0.034), and necrotic effects were not detected. Percentages of apoptotic and necrotic cells were not significantly modified by treatment with BPAF at any of these doses.

**Table 1:** Results of apoptosis/necrosis assay in primary human osteoblast lines treated for 24 h with BPS, BPF, or BPAF at doses of 10^–5^, 10^–6^, and 10^–7^ M

	% Necrosis			% Apoptosis			% Viable cell		
	Media	SD	*p*	Media	SD	*p*	Media	SD	*p*
Control	2.63	1.069	–	7.6	1.375	–	89.767	1.858	–
S3	1	0.1	0.117	26.6	4.453	0.000*	72.4	4.424	0.000*
S4	1	0.173	0.115	12.567	0.666	0.048*	86.433	0.265	0.141
S5	1.73	0.503	0.283	12.867	2.248	0.039*	85.403	2.663	0.092
F3	1.3	0.265	0.064	12.6	0.603	0.034*	86.1	0.850	0.064
F4	1.1	0.520	0.039	7.933	2.926	0.869	90.967	2.689	0.161
F5	1.833	0.907	0.233	8.7	3.477	0.589	89.467	4.331	0.929
AF3	0.933	0.351	0.099	6.633	1.106	0.411	92.411	0.709	0.052
AF4	0.967	0.115	0.113	7.833	1.790	0.839	91.2	1.955	0.270
AF5	0.867	0.306	0.094	8.533	1.070	0.427	90.6	0.781	0.501

*SD* standard deviation. *p < 0.05

### Effect of BPF, BPS, and BPAF on the synthesis of alkaline phosphate

**Fig. 2:**
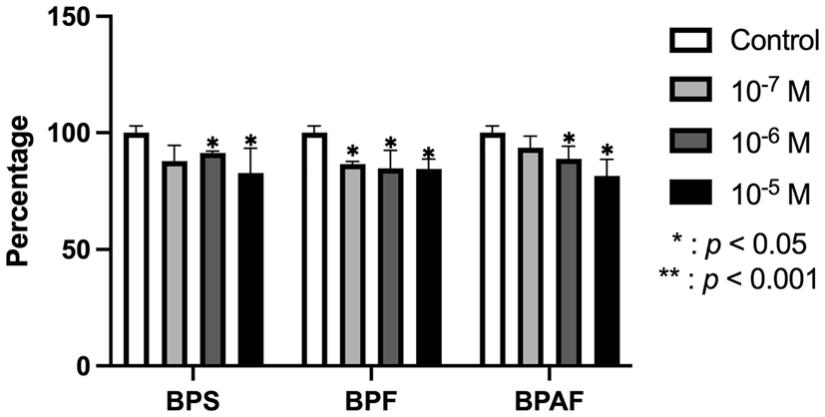
ALP activity of primary cell line after 24 h of treatment with BPS, BPF, or BPAF at doses of 10^–5^ M, 10^–6^ M, or 10^–7^ M. Activity was measured in cell lysates and normalized to total cellular protein (U/mg protein). Data are reported as means ± standard deviation

Figure 2 depicts the ALP activity of human osteoblasts at 24 h of treatment with doses of 10^–5^, 10^–6^, and 10^–7^ M of BPF, BPS, or BPAF. As observed, treatment with BPF inhibited enzymatic activity at the three studied doses. However, treatment with BPS and BPAF only produced significant changes at the higher doses (10^–5^ and 10^–6^ M).

### Effect of BPF, BPS, and BPAF on mineralization in vitro

**Fig. 3:**
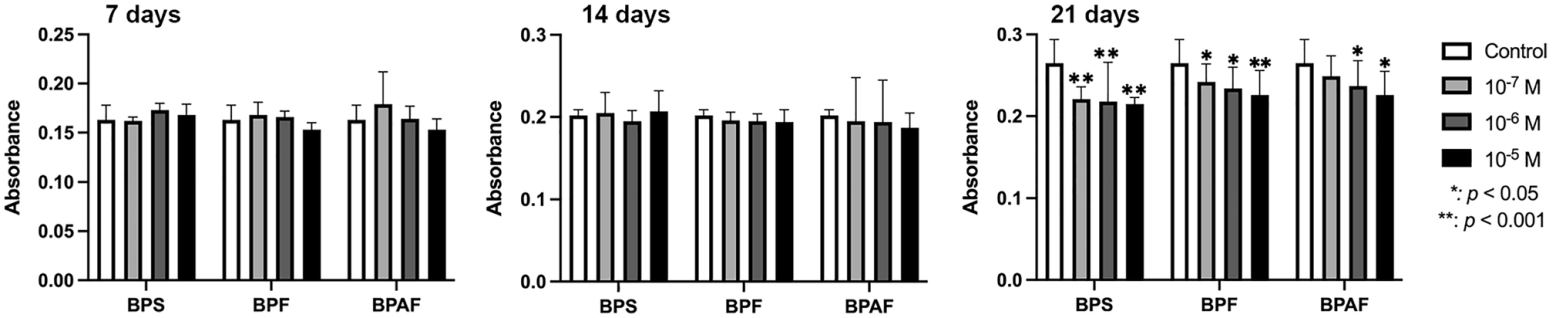
Quantitative study of mineralization (nodule formation) after culture of primary osteoblast line in osteogenic medium supplemented with BPS, BPF, or BPAF (10^–5^ M, 10^–6^ M, or 10^–7^ M). Absorbance data are reported as means ± standard deviation

Mineralization was measured at 7, 14, and 21 days of culture in osteogenic medium culture supplemented with BPF, BPS, or BPAF at doses of 10^–5^, 10^–6^, or 10^–7^ M. The results depicted in Fig. 3 show no significant change in mineralization *versus* controls at 7 or 14 days of treatment but a significant inhibition at 21 days, due to a reduced formation of calcium nodules with all three doses of BPF or BPS and the higher doses of BPAF (10^–5^ and 10^–6^ M).

## Discussion

BPA analogs have widely replaced BPA as apparently non-toxic alternatives in the fabrication of utensils and containers, especially in the food industry. However, their innocuous status is currently under debate. In this study of cultured human osteoblasts, BPA analogs BPS, BPF, and BPAF were found to adversely affect their physiology, altering their growth, ALP synthesis, and mineralization in a dose-dependent manner.

According to the present findings, the proliferation of cultured human osteoblasts is significantly inhibited by 24 h of treatment with BPS at doses of 10^–5^, 10^–6^, or 10^–7^ M and with BPF at the highest dose (10^–5^ M), and this inhibition is related to their induction of apoptosis*.* Treatment with BPAF at the same doses had no effect on the growth of this cell population. Our research group previously reported that BPA itself (at doses of 10^–5^, 10^–6^, or 10^–7^ M) inhibits the proliferation of cultured human osteoblasts by inducing apoptosis (García-Recio et al. 2022), in agreement with observations of its adverse effect on the growth of mouse (Hwang et al. 2013) and human fetal (Thent et al. 2018) osteoblasts (MC3T3-E1 and hFOB1.19 lines, respectively).

Treatment with each analog was found to dose-dependently inhibit ALP synthesis and mineralization, altering the differentiation and function of this cell population. The same inhibitory effects were reported in mouse osteoblasts treated with BPA at doses of 2.5 and 12.5 µM but not 0.5 µM (Hwang et al. 2013) and in BPA-treated human osteoblasts at even the lowest doses tested (10^–6^ and 10^–7^ M) (García-Recio et al. 2022).

It has been demonstrated that both BPS and BPF can bind to estrogen receptors and exert similar estrogenic and antiandrogenic activities to those of BPA, with BPF having less potent effects in comparison to BPA or BPS. Accordingly, both BP analogs have evidenced steroidogenic activity, teratogenicity, genotoxicity, carcinogenicity, and metabolic effects (Rosenmai et al. 2014; Eladak et al. 2015) also observed the negative repercussions of BPS and BPF on the biological function of humans and rodents, and Rochester and Bolden (2015) described the toxicity of BPS as equivalent to that of BPA. Since then, in vitro and in vivo studies have verified the adverse effects of BPS exposure on reproductive, endocrinal, and nervous systems in animals and humans, which may even include the triggering of oxidative stress (Eladak et al. 2015; Rochester and Bolden 2015; Qiu et al. 2016; Boucher et al. 2016a; Zhao et al. 2017; Wu et al. 2018). BPF exposure has adverse health effects via similar mechanisms to those observed for BPA and BPS, altering signaling pathways involved in lipid metabolism and adipogenesis (Boucher et al. 2016a, b; Lehmler et al. 2018) and causing DNA damage (Cabaton et al. 2009) Lehmler et al. 2018. However, it was recently found that neither BPA nor BPA impairs the viability or differentiation of cultured murine osteoclasts (RAW 264.7 line) at doses of 0.1, 1, or 10 µM (Kim et al. 2021).

Likewise, in vitro and in vivo research findings have shown that BPS and BPF have a similar metabolism and biological fate to that of BPA (Rosenmai et al. 2014; Rochester and Bolden 2015). Food is currently considered the predominant source of human exposure to BPS, with a relatively small contribution from personal care products. BPS and BPF are detected in urine (but rarely in other human matrices) at a lower concentration than that of BPA, which may be attributable to the longer time period of exposure to BPA (Wu et al. 2018; Lehmler et al. 2018). Although the percutaneous absorption of BPS is lesser, its lower biotransformation efficiency results in a plasma clearance rate *two-fold lower than* that of BPA. This negative effect should be considered when evaluating the consequences of replacing BPA with BPS (Liu and Martin 2019; Gayrard et al. 2020). The above evidence suggests that the presence of these analogs in the blood may have the same consequences for bone tissue as observed for other tissues and systems.

The health effects of exposure to BPAF are less well-known. It has been found to increase oxidative stress in erythrocytes to an even greater degree than observed after BPA or BPS treatment (Maćczak et al. 2017; Huang et al. 2020). In the same way, BPAF proved to be a more potent endocrine disruptor than BPA in in vivo studies of different species, such as zebra-fish and rat (Yang et al. 2016; Li et al. 2016) and was found to compromise the reproductive health of mice, in both in vitro and in vivo studies (Liang et al. 2017; Siracusa et al. 2018).

On the other hand, the observed effect of BPA analogues on osteoblasts proliferation and differentiation and/or maturation is closely related to the effect recently described by (García-Recio et al. 2023), when analyzing the modulation of osteogenic markers in the presence of these BPs.

Molecular studies have attributed differences in toxic effects among different bisphenols (BPA, BPAF, and BPS) to variations in their affinities and binding sites. Multiple sites with variable binding affinities have been described in the androgen receptor for these bisphenols, indicating the availability of modified binding surfaces on this receptor for co-regulating interactions (Perera et al. 2017).

In the present study of human osteoblasts, BPF and BPS affected both their growth and differentiation, while BPAF altered their differentiation alone, through the dose-dependent inhibition of ALP synthesis and mineralization. These data suggest that BPA analogs can impair bone health, although further research is needed to verify these effects. Our results are in line with the need to reduce the intake of BPs, as stated by EFSA in its latest report (EFSA 2021). And on the other hand, the use of these BPs in industry, given that an increase in the presence of BPs, including those studied in this work, has been observed in the environment (Liu et al. 2021; Catenza et al. 2021).

According to these results, BPF and BPS adversely affect the viability, differentiation, and function of cultured human osteoblasts in a dose-dependent manner, whereas exposure to BPAF only alters their ALP synthesis and mineralization.

## Data Availability

The datasets generated during and/or analysed during the current study are available from the corresponding author on reasonable request.
